# X-ray nanoimaging of a transversely embedded carbon fiber in epoxy matrix under static and cyclic loads

**DOI:** 10.1038/s41598-022-12724-1

**Published:** 2022-05-25

**Authors:** Kosuke Takahashi, Ryosuke Shoya, Takuma Matsuo, Wataru Sato, Takashi Nakamura, Akihisa Takeuchi, Masayuki Uesugi, Kentaro Uesugi

**Affiliations:** 1 Division of Mechanical and Aerospace Engineering, Faculty of Engineering, Hokkaido University, Sapporo, Japan; 2 Department of Mechanical and Space Engineering, Graduate School of Engineering, Hokkaido University, Sapporo, Japan; 3 Japan Synchrotron Radiation Research Institute (JASRI)/SPring-8, Sayo District, Japan

**Keywords:** Mechanical engineering, Imaging techniques

## Abstract

The initial stage of fatigue failure has not been thoroughly clarified for carbon fiber reinforced plastics (CFRPs). Although the initiation of fatigue cracks has been regarded to be interfacial debonding between the carbon fiber and polymer matrix, their detection among numerous carbon fibers, whose diameter is only 7 µm, is extremely difficult. In this study, a single carbon fiber was transversely embedded in a dumbbell-shaped epoxy sample to focus on the interfacial debonding and was observed using synchrotron radiation (SR) X-ray computed tomography (CT). A tabletop fatigue testing machine driven by a piezoelectric actuator was developed to apply static and cyclic loads along the beamline. SR X-ray multiscale CT imaging was conducted by switching between an absorption-contrast projection method (micro-CT) and a phase-contrast imaging-type X-ray microscopic CT (nano-CT). The carbon fiber was entirely captured by micro-CT and then magnified at both ends on the free surfaces. Nano-CT clearly visualized the interfacial debonding under 30 MPa static tensile load and the implication of the coalescence of nano-voids along the interface under 50 MPa. Under cyclic loads, the interfacial debonding gradually progressed under a 8–40 MPa sinusoidal stress after 10,000 cycles, whereas it did not propagate under a stress below 30 MPa.

## Introduction

Carbon fiber reinforced plastics (CFRPs), which have high specific stiffness and strength with their high tunability^1^, are expected to drive considerable progress particularly in aerospace engineering. Innovative manufacturing techniques of CFRPs are also being proposed as next-generation fabrication methodology^2,3^. However, the CFRP structures have been still designed conservatively because of the difficulty in the safety assessment as the inhomogeneity of the material causes complicated fracture behaviors, including fiber breakage and delamination^4^. In particular, the initiation of fatigue cracks is not well understood for CFRP structures although fatigue failure accounts for 70% of the causes of mechanical failures. Thus, fatigue tests, despite their high costs, must be conducted for different laminate configurations and applied stress levels. Several failure assessments based on phenomenological approaches have been proposed^5–8^, but they are not specifically related to the mechanical properties of the carbon fiber, polymer matrix, and their interface. Therefore, a versatile evaluation method based on the general mechanism of the initiation and propagation of the fatigue crack in CFRP structures is desired.

One key aspect of fatigue failure is the transverse crack generated in the 90° layers, where carbon fibers do not directly affect the strength in the loading direction. The generation of the transverse crack is regarded to be the first process of fatigue failure as it gradually increases during the early stage of the loading cycles^9–12^. Although transverse cracks do not immediately degrade the rigidity and strength of CFRP laminates, they eventually cause delamination between layers with different fiber orientations. Hosoi et al. proposed an estimation method for the remaining fatigue life based on the accumulation of transverse cracks^13–15^; however, predicting the initiation of a transverse crack remains a considerable challenge because it strongly depends on the laminate configurations.

**Figure 1:**
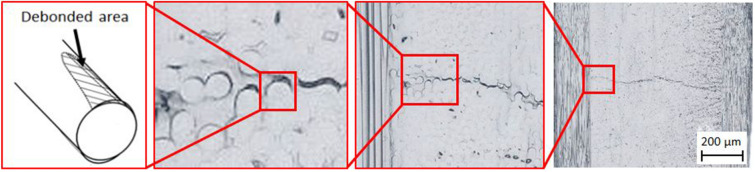
Magnified image of a transverse crack in a cross-ply laminate implying the initiation of interfacial debonding between the carbon fiber and epoxy matrix. The transverse crack resulted from the propagation of the matrix cracking, which was caused by connecting the interfacial debonding of neighboring carbon fibers.

As the origin of a transverse crack, the interfacial debonding between the carbon fiber and polymer matrix has been previously studied^16^, as shown in Fig. 1. The observation at the site of the transverse crack using a scanning electron microscope also implied the propagation along the interface of the carbon fibers through matrix cracking^13^. However, detecting the nanoscale opening gap of the interfacial debonding among the innumerable carbon fibers in the CFRP laminate before it propagates to become a transverse crack is extremely difficult. Although finite element analysis considering the interfacial debonding in addition to yielding and cracking of the polymer matrix has been utilized to clarify the formation of transverse cracks, it usually requires fitting parameters to represent the experimental results^17–20^.

The interfacial property of a specific carbon fiber can be characterized using a single fiber fragmentation test by embedding a single carbon fiber into the polymer matrix. Although the carbon fiber is generally embedded along the loading direction to measure the interfacial shear strength^21–23^, the characterization of interfacial debonding in the transverse direction was also proposed as a cruciform specimen method^24^. This method observes the interfacial debonding initiated from the interior of the matrix resin; however, it may not correspond to those initiated from the CFRP laminates because transverse cracks are generally initiated from the free surface. Martyniuk et al. prepared an epoxy sample embedding a relatively large single glass fiber with a diameter of 50 µm, and observed it using synchrotron radiation (SR) X-ray computed tomography (CT)^25^. The three-dimensional (3D) debonding behavior of the glass fiber was confirmed from the free surface toward the interior of the sample. This suggests that the interfacial debonding of a carbon fiber could also be observed, but existing studies of SR X-ray CT applied to CFRPs are at a sub-microscale, which is not sufficient to visualize the interfacial debonding of carbon fibers, whose diameter is only 7 µm^26–31^.

In this study, an epoxy sample with a single carbon fiber embedded in the transverse direction was prepared to capture the interfacial debonding using advanced technology of SR X-ray CT at the large SR facility SPring-8 (Hyogo, Japan). To realize the in-situ SR X-ray CT, a piezoelectric actuator-driven desktop fatigue testing machine was developed to directly apply static and cyclic loads along the beamline. The carbon fiber was observed under static and cyclic loads to capture the initiation and propagation of interfacial debonding.

## Materials and methods

### Sample preparation

The epoxy matrix used in this study comprised jER828 and the curing agent jER Cure 113 (Mitsubishi Chemical) at a weight ratio of 100:32. A relatively hard curing agent was selected to minimize the viscoelastic effect during loading. They were mixed using a vacuum defoaming stirrer VDS-1 (ASONE Corporation) under vacuum for 5 min and completely degassed in a vacuum oven DRV320DB (Advantec Toyo Kaisha, Ltd.) preheated at 80 °C for 15 min. Subsequently, a single carbon fiber T700SC (Toray Industries, Inc.) was placed across the parallel part of the silicon mold. Weights of 1 g each were attached to both ends to prevent fiber waviness due to the curing shrinkage of the epoxy. The vacuum-defoamed epoxy was then poured into the silicon mold. The curing cycle was first conducted at 80 °C for 1 h and then 150 °C for 3 h. The surfaces of the cured samples were carefully polished with emery paper in the order of #1000, #1500, and #2000 and then mirror-finished with a liquid compound with grain sizes in the order of 7, 1, and 0.2 μm.

**Figure 2:**
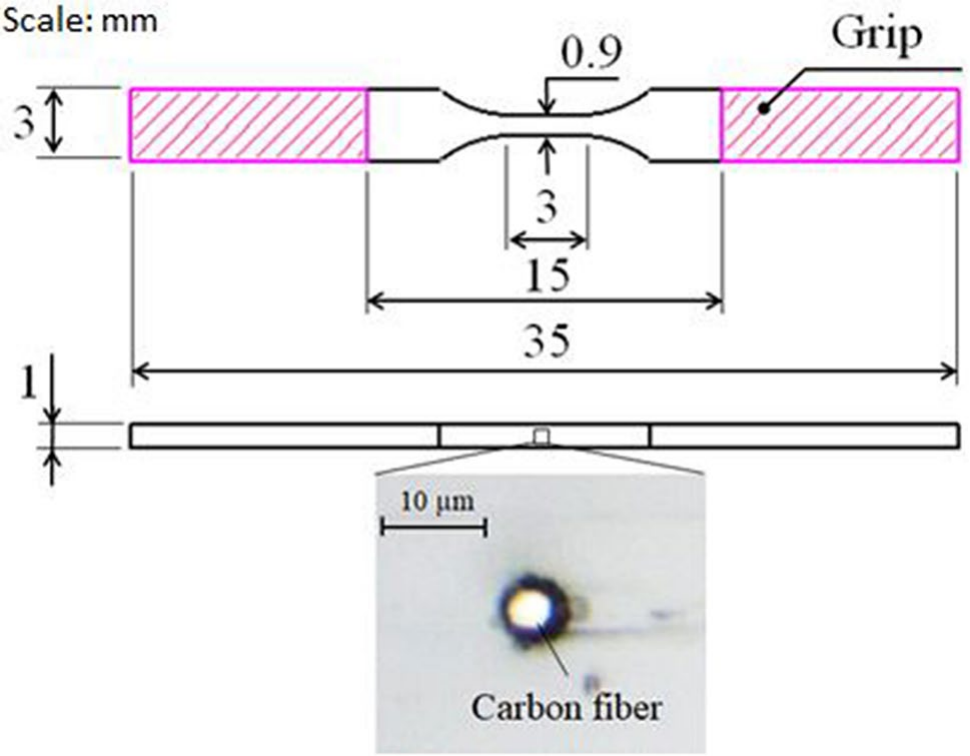
Dimensions of a dumbbell-shaped epoxy sample with a single carbon fiber embedded in the transverse direction. An image of a single carbon fiber observed under a high-magnification digital microscope.

The dimension of the sample as well as magnified image of the carbon fiber observed using a digital microscope MS-300 (Asahi Kougakuki Manuf. Co., Ltd.) is shown in Fig. 2. The dark boundary zone of the carbon fiber is the shade because it slightly protruded from the epoxy surface after the polishing process due to their difference in hardness. The yield stress (0.5% proof stress), tensile strength, and elastic modulus of the neat epoxy sample were 60.4 MPa, 106.3 MPa, and 3.12 GPa, respectively, measured by a tensile test.

### SR X-ray multiscale CT

**Figure 3:**
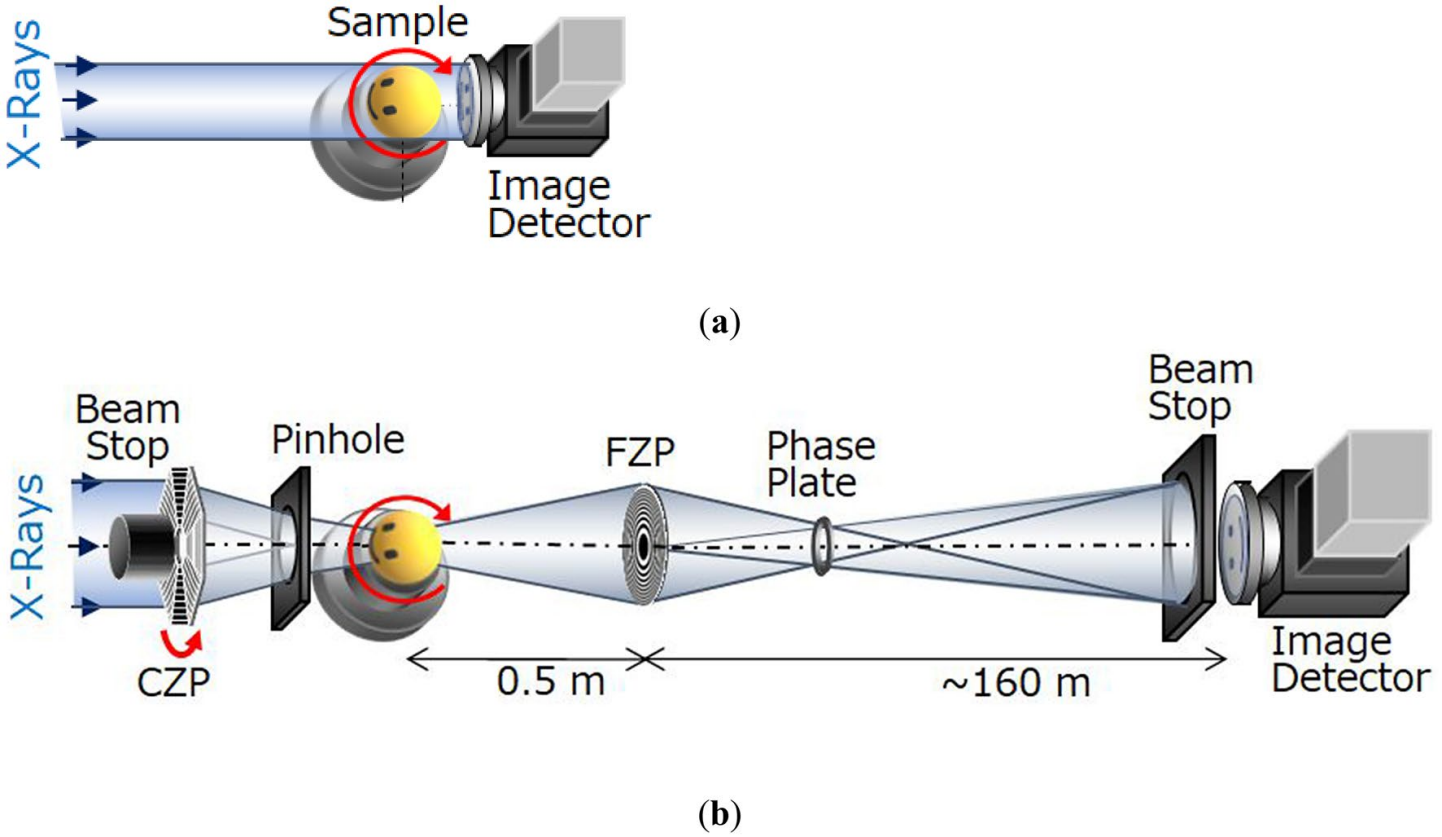
Schematics of (**a**) micro-CT and (**b**) nano-CT as synchrotron radiation multiscale X-ray CT available at beamline 20XU of SPring-8^32^. In micro-CT, a quasi-parallel beam transmitted through the rotating sample is captured with an image detector installed immediately behind the sample. Nano-CT comprises a hollow-cone beam illumination system with a condenser zone plate (CZP), sample stages, a Fresnel zone plate (FZP) as an objective, and an image detector installed in a separate experimental hutch located 160 m away.

Figure 3a shows a schematic of the absorption-contrast projection method called “micro-CT”^32^. In detail, a sample is placed on a rotating stage and irradiated with monochromatic X-rays. The transmitted X-ray image is detected by a visible-light conversion type X-ray camera consisting of a scintillator, optical lens, and complementary metal-oxide semiconductor camera. Therefore, the spatial resolution depends on the detector specifications, which is generally at a sub-microscale.

In recent years, a high-resolution X-ray microscopic CT, called “nano-CT”, has been developed to further improve the spatial and density resolutions of conventional X-ray CT methods^33^. Figure 3b shows a schematic of the nano-CT system. The X-ray transmitted through the sample is magnified using the Fresnel zone plate, which is an objective lens, to produce the image on the detector with improved spatial resolution. In addition, introducing the phase-contrast imaging method emphasizes the slight differences in the density to distinguish the carbon fiber from the epoxy matrix^34,35^. Phase-contrast imaging utilizes the phase shift when X-ray pass through an object, whereas conventional X-ray absorption-contrast imaging is based on the change in the intensity. The sensitivity of the phase shift is three orders of magnitude greater than that of change in the intensity^36^.

At SPring-8, a combination of micro-CT and nano-CT systems, called multiscale CT, is available along the beamline BL20XU. These two systems can be easily switched by keeping the position of the sample on the rotational stage. In detail, micro-CT is conducted to specify the region of interest (ROI) from the entire structure of the sample, after which nano-CT is conducted to focus on the ROI. This multiscale CT system is particularly beneficial for the nondestructive inspection of tiny damages. The field of view and pixel size of the micro-CT and nano-CT systems are 1 mm^2^ with 495 nm/pixel and 62.5 µm^2^ with 42 nm/pixel, respectively. In this study, the X-ray energy was set to 20 keV, and the exposure time per irradiation was 0.1 s for micro-CT and 0.5 s for nano-CT. The sample was rotated from 0° to 180° in 0.1° increments during X-ray irradiation, which required approximately 3 and 15 min for micro-CT and nano-CT, respectively.

**Figure 4:**
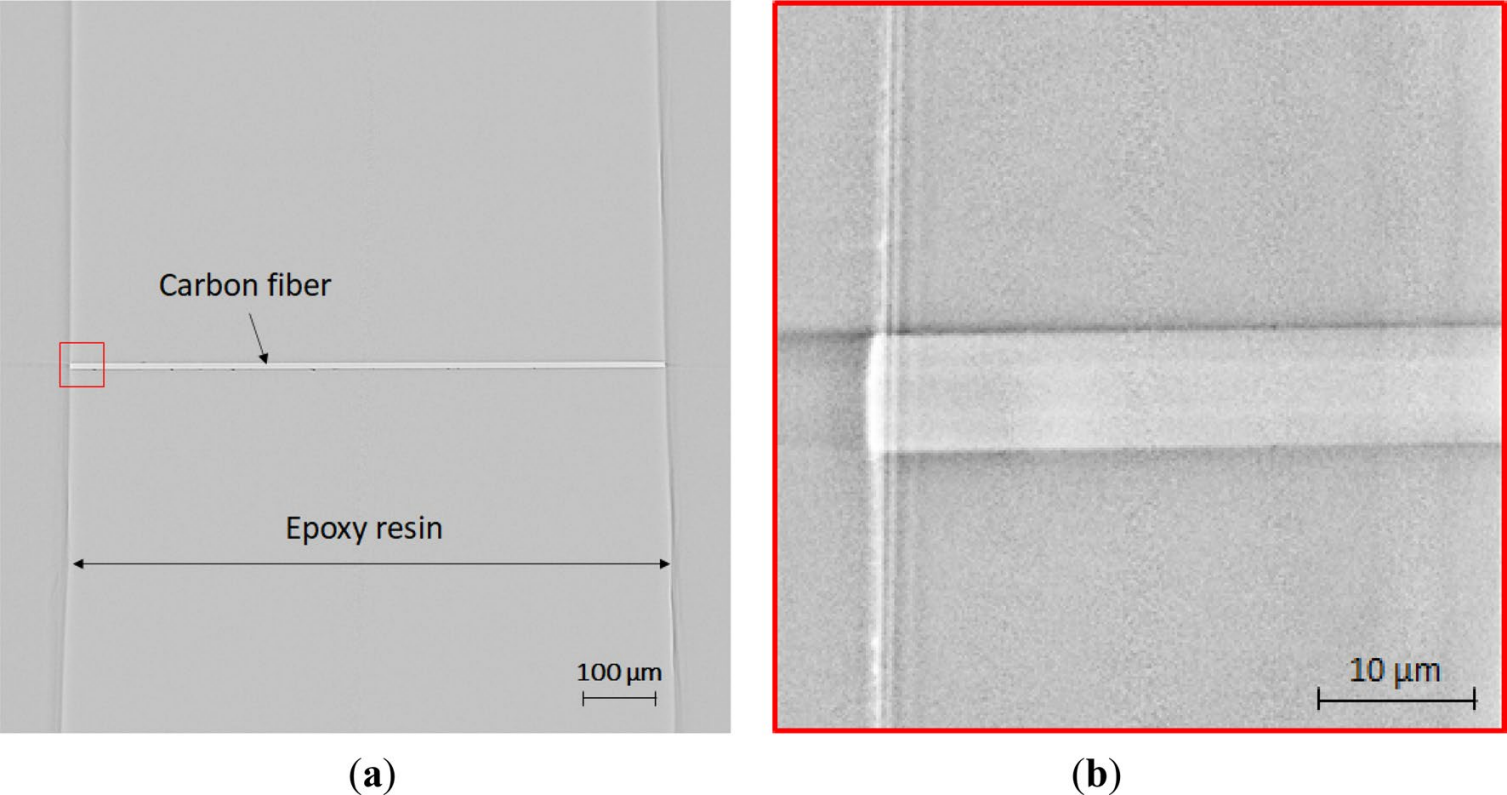
Reconstructed images along the longitudinal cross-section by (**a**) micro-CT and (**b**) nano-CT. The carbon fiber was entirely captured by micro-CT and then focused by nano-CT in the red square.

Examples of the micro-CT and nano-CT images of a single carbon fiber embedded at the center of the epoxy sample are shown in Fig. 4a,b, respectively. These are the re-sliced views along the fiber diameter from the 3D reconstructed image using 1800 transmission images. The slight difference in the density between the carbon fiber and epoxy matrix can be distinguished by both micro-CT and nano-CT. All the cross-section of the carbon fiber was captured by micro-CT. The end of the carbon fiber was magnified using nano-CT, which corresponds to the red square region in the micro-CT image. The image before loading confirmed that the carbon fiber is closely bonded to the epoxy matrix.

### Development of the piezoelectric actuator-driven tabletop fatigue testing machine

The fatigue test is generally conducted using a heavy hydraulic testing machine, which must be operated away from the beamline because of its potential to cause unwanted vibrations. Therefore, it was necessary to remove the sample from the fatigue testing machine and replace it in the rotation stage of the beamline for CT imaging. This process consumed user time at the synchrotron facility and also increased the risk of accidental damage to the sample.

**Figure 5:**
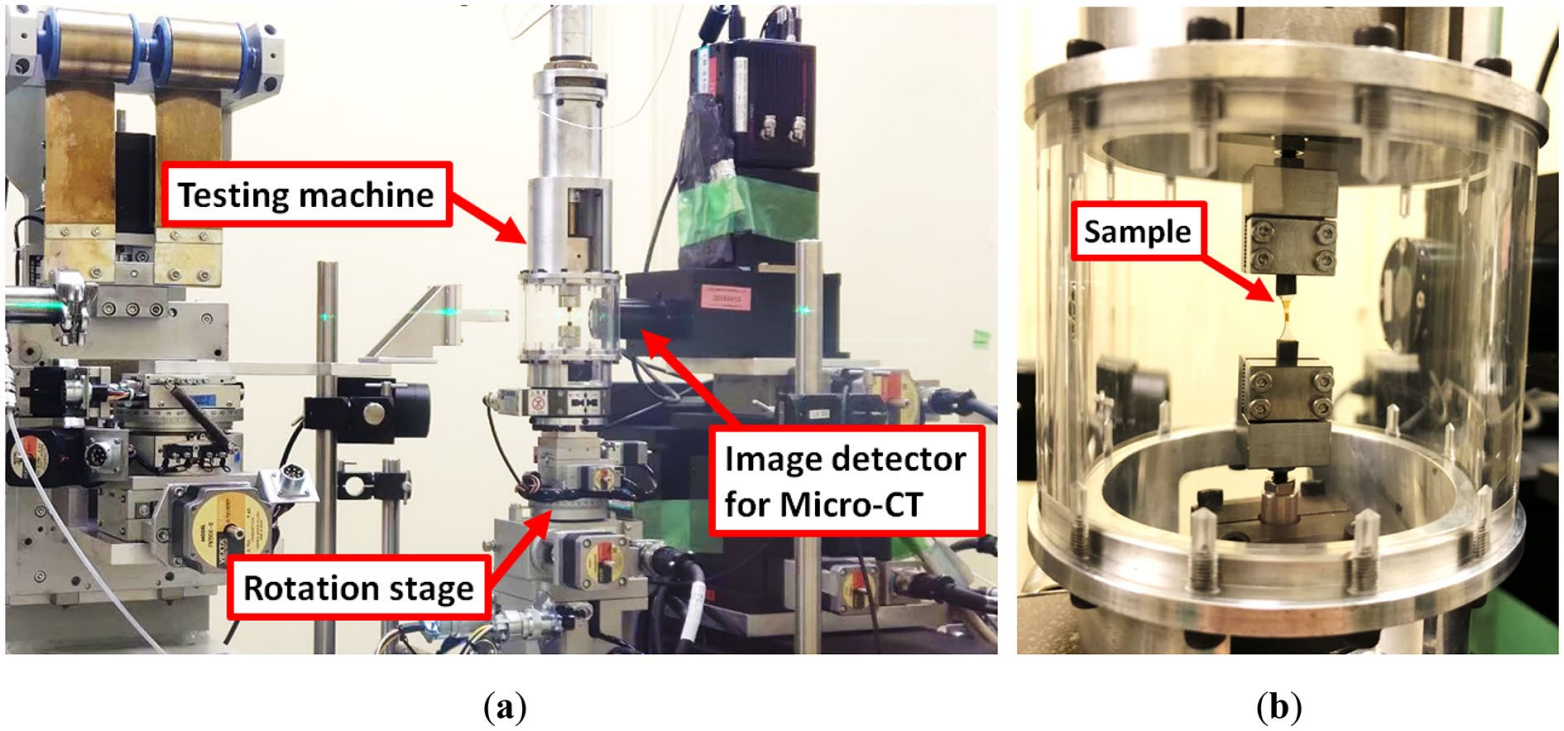
(**a**) Tabletop fatigue testing machine driven by a piezoelectric actuator installed on the beamline 20XU at SPring-8 and (**b**) magnified image around the clamped sample.

For more efficient in-situ observations by SR X-ray CT, we developed a tabletop fatigue testing machine that allows the application of both static and cyclic loads on the beamline^37,38^. Our testing machine allows small increments of loading cycles to capture the gradual propagation of the internal damage by CT imaging. The testing machine mounted on the rotation stage of the beamline is shown in Fig. 5a. The total weight is less than 3 kg because a piezoelectric actuator PSt 150/10/200 VS15 (Piezomechanik Co., Ltd), which allows a stroke of 270 µm and a static tensile load of 600 N, was employed. A load cell was connected to a clamp fixed to the bottom of the testing machine. The clamped sample was surrounded by an acrylic cylinder with a thickness of 3 mm through which the X-rays were transmitted, as shown in a magnified view around the sample in Fig. 5b.

### In-situ observation of interfacial debonding between the carbon fiber and epoxy matrix

**Figure 6:**
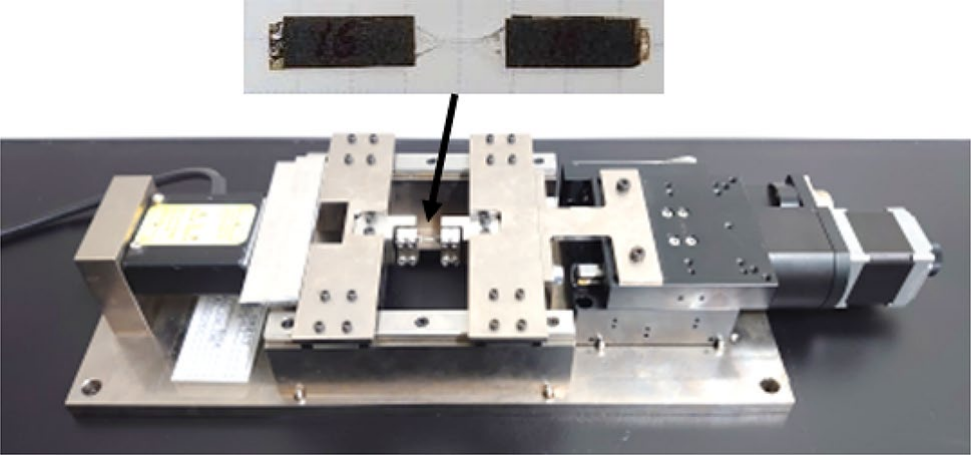
Tabletop tensile testing machine for the in-situ observation of the sample under an optical microscope.

The tensile stress required to cause interfacial debonding between the carbon fiber and epoxy matrix was first investigated by the stepwise increase of the tensile load under repeated loading and unloading. A tensile load was applied using a small tensile testing machine developed in our laboratory (Fig. 6) for the in-situ observation under a high-magnification digital microscope. The sample was loaded to 10 MPa and unloaded to 5 MPa at a tensile velocity of 10 µm/s. The same process was repeated by adding 10 MPa for the next loading process until fracture.

Based on the preliminary test results, the carbon fiber was observed using SR X-ray CT under a static tensile load applied by the developed fatigue testing machine. The carbon fiber was entirely captured using micro-CT and then focused at both ends on the sample surfaces using nano-CT under constant stress. Different values of tensile stress were applied to clearly capture the interfacial debonding. Because repeated X-ray irradiation could damage the epoxy matrix, the samples were individually prepared for each stress condition.

The fatigue test was also conducted along the beamline. The maximum stress was determined based on the static tensile test. Accordingly, the piezoelectric actuator was controlled such that the stress ratio *R* = 0.2. The frequency of the cyclic loading was set to 10 Hz for up to 10,000 cycles and 20 Hz thereafter. The displacement was maintained at the mean value of the cyclic loading during the imaging process. The fatigue test requires repeated imaging of the same sample to track the propagation of the interfacial debonding; however, excessive exposure to X-rays may damage the epoxy matrix. Therefore, to minimize the radiation damage to samples during the fatigue test, simple X-ray transmission images obtained by a single shot exposure was used to evaluate the interfacial debonding, rather than the CT images requiring 1800 shots of exposure. The X-ray transmission images were taken at a position where the tip of the carbon fiber can be observed.

## Results and discussion

### In-situ observation under a digital microscope for the loading and unloading test

**Figure 7:**
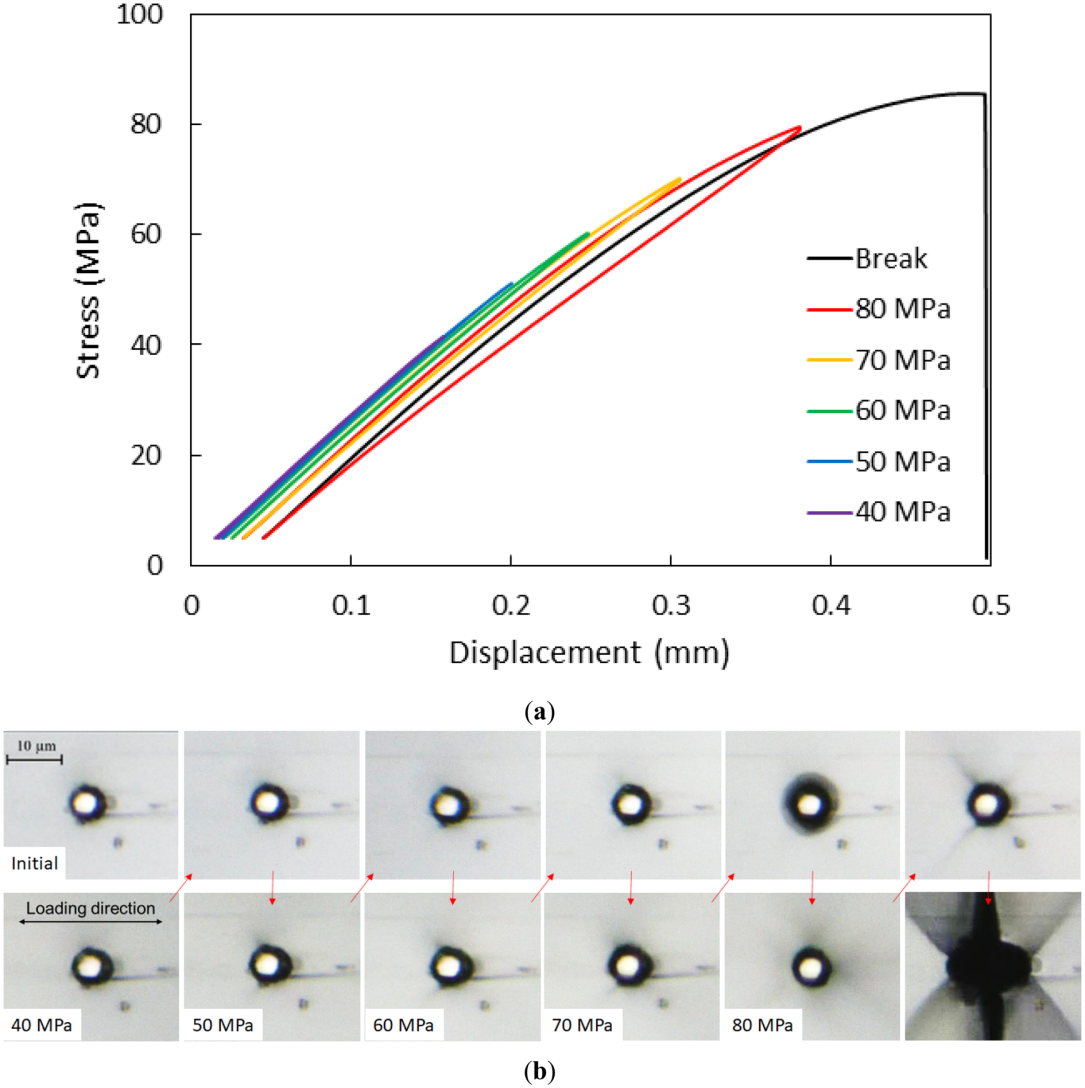
(**a**) Loading–unloading curve of the epoxy sample embedded with a single carbon fiber. The gradual increase in residual displacement after unloading implies the progress of the interfacial debonding. (**b**) Optical images observed under high-magnification microscope before and after unloading with an increment of 10 MPa until fracture. The lower and upper images were captured before and after unloading at each loading cycle, respectively. The dark boundary zone became gradually thicker at the higher loadings, and confirming the interfacial debonding by observing the sample surface was difficult.

The results of the loading–unloading test are shown in Fig. 7a. The vertical axis represents the nominal stress of the cross-sectional area at the parallel part of the sample. The horizontal axis represents the displacement between the grips measured by a laser displacement meter because the sample was too small to attach a strain gauge. The loading and unloading curves below 30 MPa did not show any hysteresis or change; thus, they are omitted here. The small residual displacement was confirmed after unloading from 40 MPa, and it gradually increased after unloading from subsequent loadings. The sample was fractured before reaching 90 MPa.

The optical images observed under the high-magnification microscope around the carbon fiber are shown in Fig. 7b. Comparing the image before loading (upper left), the boundary of the carbon fiber on the right side became slightly thicker at 40 MPa, but returned to look similar after unloading to 5 MPa. The dark boundary zone appeared to be thicker at the subsequent higher loadings, and the surrounding area became shady after unloading from 70 MPa. The surrounding shady area disappeared at the loading at 80 MPa, but after unloading the local shear bands appeared in the 45° angled from the loading direction. The fracture then occurred from the boundary, but the carbon fiber did not remain there (the lower right). The center hole observed after the fracture implied the complete separation along the interface of the carbon fiber. However, it was difficult to clearly distinguish the initiation of interfacial debonding from the shade of the boundary under high-magnification digital microscope. It might be initiated above 40 MPa based on the residual displacement of the loading–unloading process.

### In-situ observation under SR X-ray CT for static tensile test

**Figure 8:**
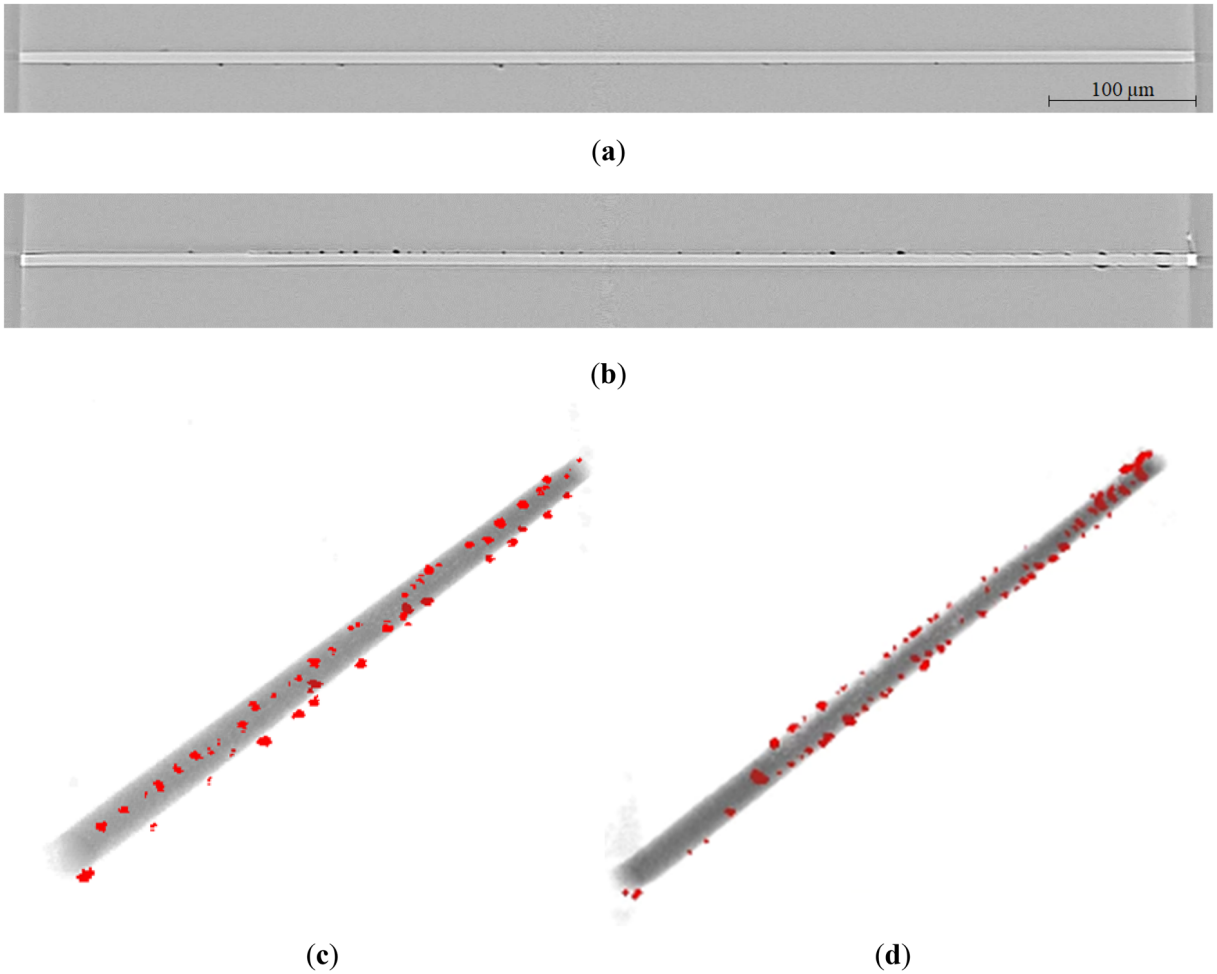
Reconstructed images obtained by micro-CT (**a**) before loading and (**b**) under a stress of 50 MPa, with the 3D image of the carbon fiber extracted using binarization process (**c**) before loading and (**d**) under a stress of 50 MPa. The empty spaces in the epoxy matrix are represented in red, and interfacial debonding was not clearly detected.

The maximum tensile stress that the developed fatigue testing machine could maintain was 50 MPa because of stroke limitations. Figure 8a–b show the result of micro-CT imaging before loading and at 50 MPa loading, respectively. They are the re-sliced views observed in the same direction as that in Fig. 4a. The voids were found randomly along the carbon fiber from the sample before loading, and specifying the interfacial debonding at 50 MPa was still difficult. The volume of the carbon fiber was then extracted from the reconstructed 3D image using binarization process. The volume of the voids (empty space) was also extracted separately and then unified with the carbon fiber, as shown in Fig. 8c–d for the samples before loading and at 50 MPa loading, respectively. The location and size of the voids, represented in red, can be clearly related to the carbon fiber; however, an indication of interfacial debonding by micro-CT was still not present.

**Figure 9:**
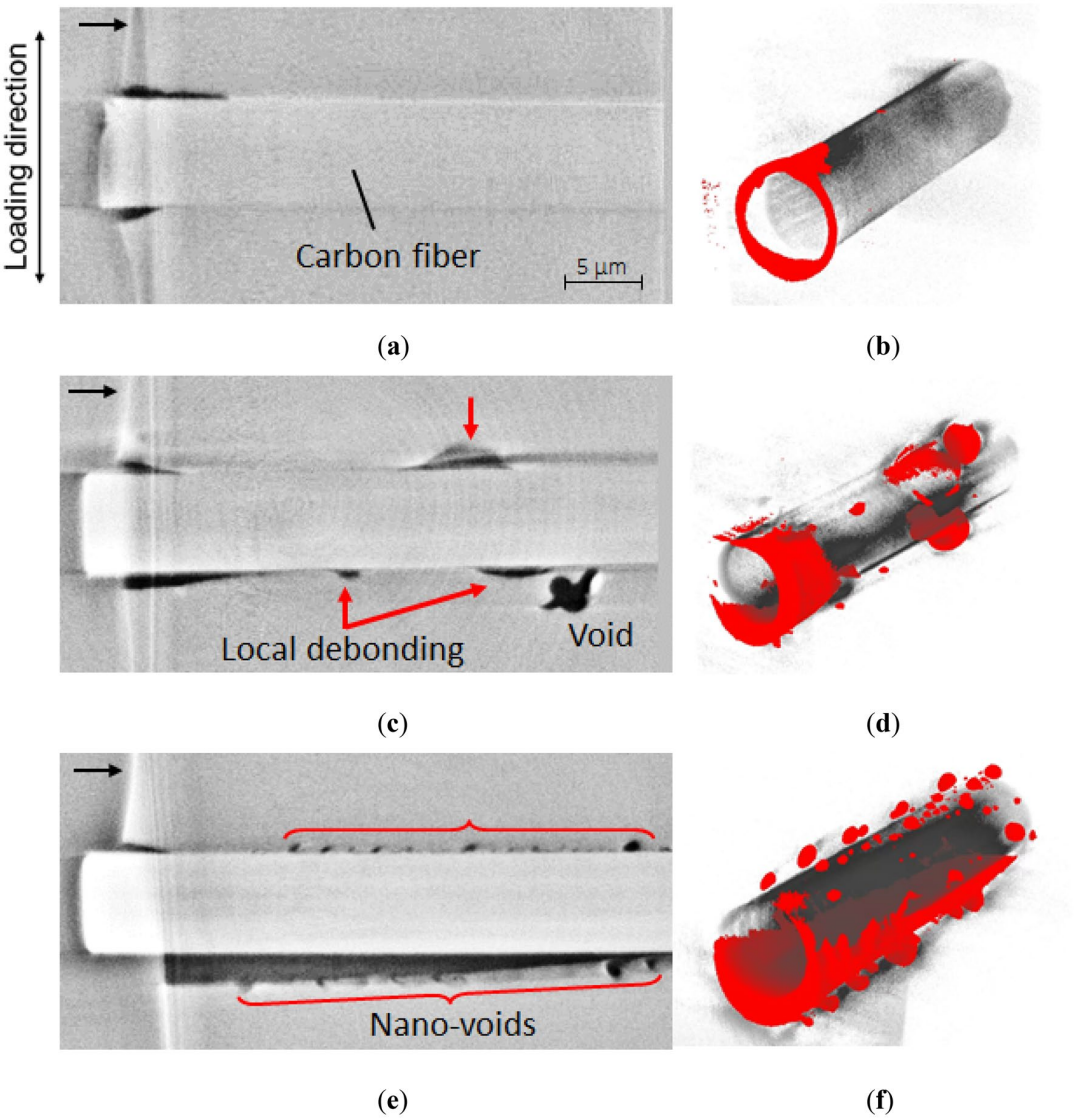
Reconstructed images recorded under stresses of (**a**, **b**) 30, **(c**, **d**) 40, and (**e**, **f**) 50 MPa by nano-CT (left) with the 3D image of the carbon fiber extracted using binarization process with empty spaces in the epoxy matrix represented in red. (right). The interfacial debonding was clearly captured from the sample surface even under 30 MPa. The local debonding isolated from the sample surface was also observed under 40 MPa. The interfacial debonding exceeded the field of view under 50 MPa, with an indication of the coalescence of voids prior to the separation from the carbon fiber.

The nano-CT images obtained under stresses of 30, 40, and 50 MPa are shown in Fig. 9a–f. It should be noted that they were different samples due to the concern of damage by X-ray. The re-sliced views observed from the same direction as that in Fig. 4b are shown on the left; the black arrow indicates the sample surface. The boundary of the carbon fiber was sufficiently clear to distinguish the interfacial debonding as the black region along the upper and lower interfaces even under a stress of 30 MPa (Fig. 9a). The volume of the carbon fiber was then extracted from the 3D reconstructed image by binarization and unified with the empty space in the epoxy matrix in red (Fig. 9b). The carbon fiber was separated from the epoxy matrix only near the sample surface but was still connected to the epoxy matrix without any voids. The interfacial debonding under a higher stress of 40 MPa was slightly longer for a larger opening, as shown in Fig. 9c–d. Local debonding isolated from the sample surface was also observed, as indicated by the red arrows. In addition, a crack was generated from the void at the lower right in Fig. 9c, which was trapped during the curing process.

When a stress of 50 MPa was applied, larger debonding along the lower interface was observed, as shown in Fig. 9e–f. From the 3D view in Fig. 9f, the lower half of the carbon fiber was almost entirely separated from the epoxy matrix. The upper side showed the tiny debonding length, but small voids were distributed along the carbon fiber. Since the size and appearance of these voids were different from those in Fig. 9c, they were not formed during the curing process, but due to the tensile load. This implies that the interfacial debonding accompanies the coalescence of these small voids, which was also proposed by Kimura et al.^34^. In addition, the debonded surface with a dimple-like texture of the epoxy matrix on the lower side exhibited void formation before separating from the carbon fiber. It was found that nano-CT has sufficient resolution and magnification to detect the interfacial debonding between the carbon fiber and epoxy matrix, and void formation prior to the debonding.

### In-situ observation under SR X-ray CT for the fatigue test

**Figure 10:**
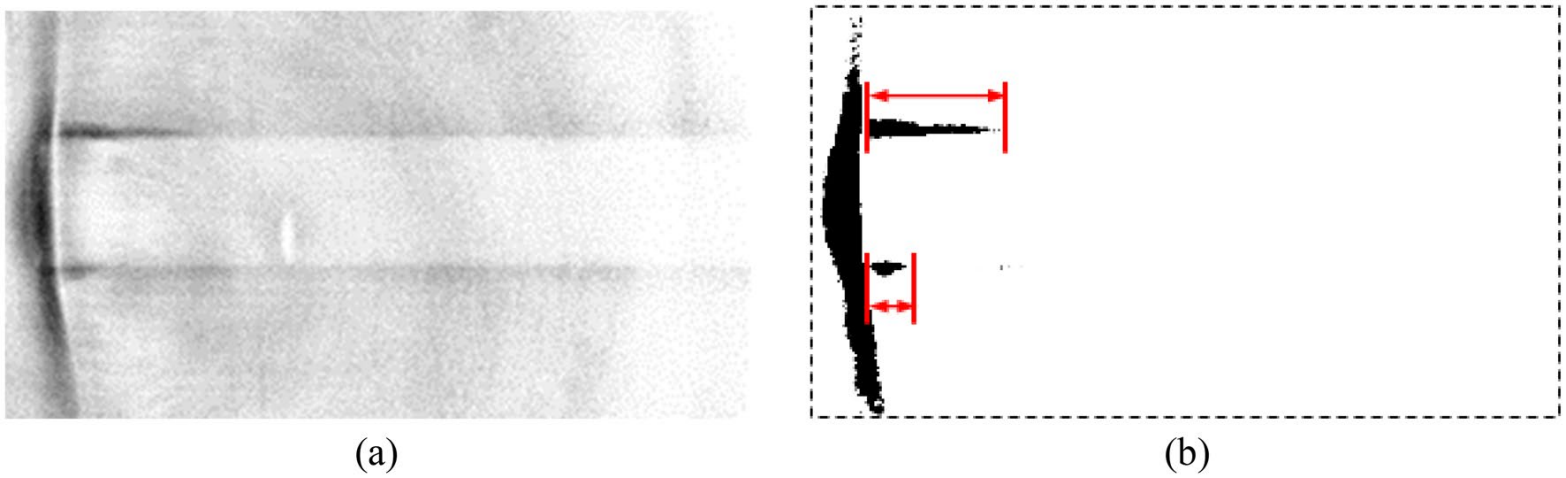
(**a**) Transmission image obtained during nano-CT imaging from the same angle as that in Fig. 9a, and (**b**) debonding length extracted using the binarization process. Distinguish the short interfacial debonding from the sample surface is still possible.

One of the transmission images, which was used for reconstructing the 3D image, from the same angle in Fig. 9a is shown in Fig. 10a. Although it was not as clear as the 3D reconstruction image of Fig. 9a, the debonding length can still be detected and tracked with an increasing the number of loading cycles. To quantitatively characterize the debonding length, the transmission image was modified using the binarization process. First, the background was adjusted to be the same brightness for all transmission images recorded at different loading cycles. The contrast was then controlled such that the boundary of the carbon fiber became invisible in the transmission image before loading. When interfacial debonding occurred, the black pixels along the carbon fiber appeared again. The binarized image of Fig. 10a is shown in Fig. 10b. The debonding length is defined as the length of clusters of black pixels represented by the red arrows, which corresponds well with the debonding length of the 3D reconstructed image in Fig. 9a.

**Figure 11:**
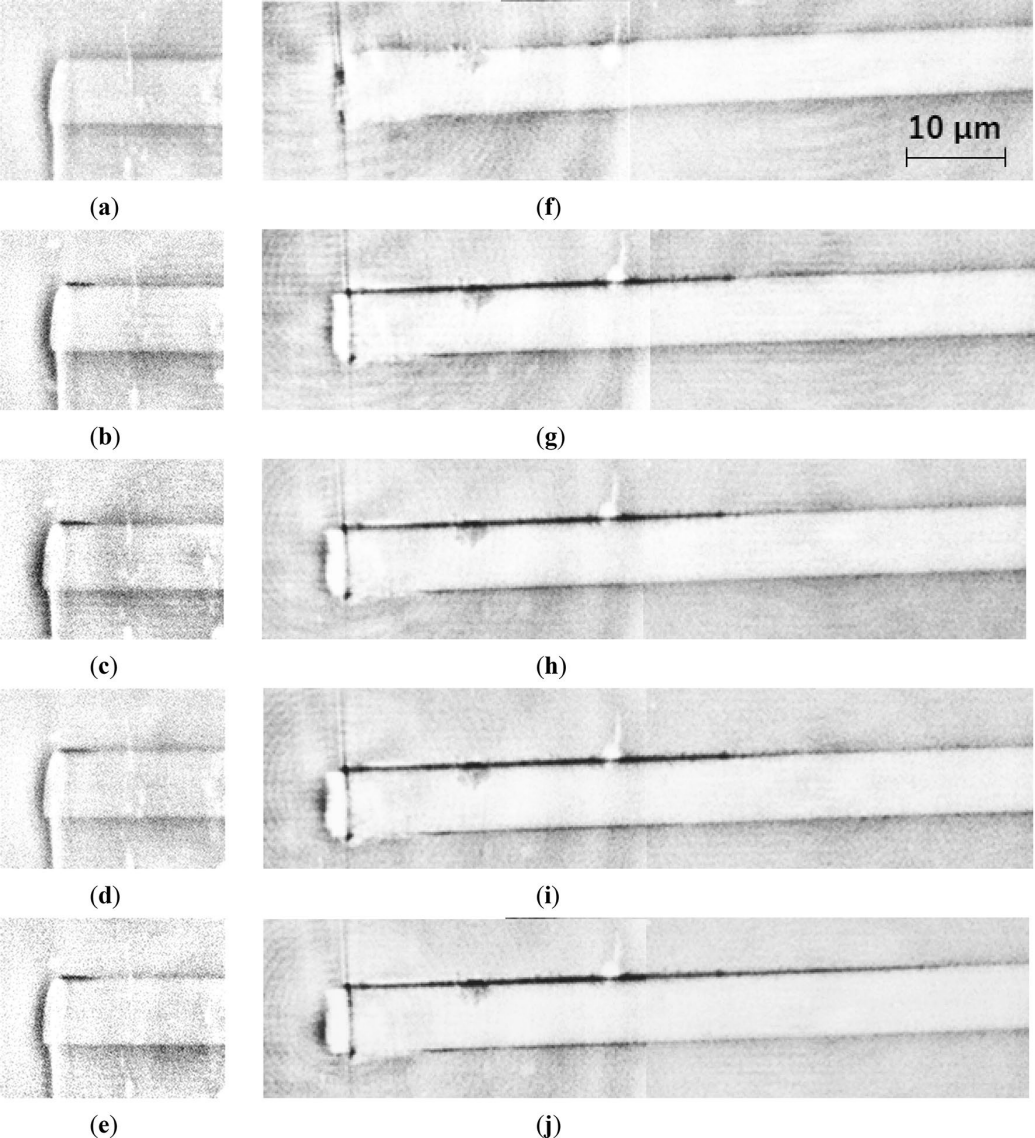
Transmission images obtained under cyclic loadings (stress ratio *R* = 0.2) with the maximum stresses of (**a**)–(**e**) 30 and (**f**)﻿–(**j**) 40 MPa. The images were captured (**a**, **f**) before loading and at (**b**, **g**) 1; (**c**, **h**) 10,000; (**d**, **i**) 30,000; and (**e**, **j**) 100,000 cycles, respectively. The interfacial debonding did not propagate under 30 MPa, but finally propagated after 10,000 cycles at the maximum stress of 40 MPa.

Based on the results of the static tensile test, a cyclic loading with a maximum stress of 30 or 40 MPa was applied. Figure 11a–e show the transmission images taken before loading and under the average stress of 6–30 MPa at 1; 10,000; 30,000; and 100,000 loading cycles, respectively. Here, the field of view was shifted to the neighboring region after recording the first transmission image at the sample surface, and two transmission images were connected to capture the entire debonding length. Although a short debonding length was observed on the upper side of the carbon fiber in Fig. 11b, the debonding did not propagate further until 100,000 loading cycles, as shown in Fig. 11c–e. The transmission images observed at the same loading cycles under the average stress of 8–40 MPa were shown in Fig. 11f–j. The interfacial debonding caused by the first cycle (Fig. 11g) did not propagate until 10,000 loading cycles, as shown in Fig. 11h, but showed a slight increase till 30,000 loading cycles finally (Fig. 11i). The debonding length continued to increase until 100,000 loading cycles (Fig. 11j).

**Figure 12:**
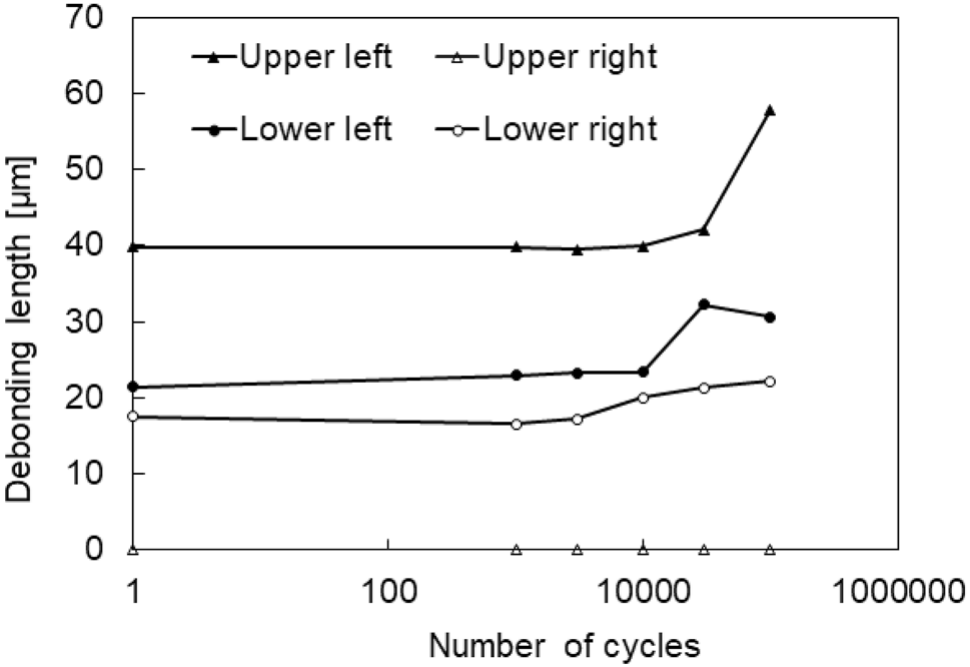
Measured lengths of interfacial debonding observed using transmission nano-imaging near the left and right sample surfaces under cyclic loadings between 8 and 40 MPa. Although the different propagation features in the left and the right ends implied that the sample was not tensioned perfectly straight, the interfacial debonding length was quantitatively captured.

The debonding lengths of the upper and lower sides on both the left and right surfaces of the sample were extracted in the same way as shown in Fig. 10b and are summarized in Fig. 12. The debonding length on the upper side of the carbon fiber was longer on the left side, whereas that on the lower side was longer on the right side. This implies that the sample was not tensioned perfectly straight but was slightly bent. Although an improved adjustment of the tension axis is required for the developed fatigue testing machine to confirm the reproducible results, the propagation of the interfacial debonding of a carbon fiber by cyclic loading was successfully captured.

The imaging quality of the clearer boundary with noise removal could be improved by optimizing the X-ray energy and applying the latest imaging techniques^39^; this will be addressed in future work. Furthermore, the interfacial debonding feature of a single carbon fiber needs to be related to the matrix cracking, which was observed from a bundle of carbon fibers embedded in the epoxy sample^37^. No indication of the matrix cracking from the single-fiber sample implies that the interaction between the interfacial debonding of neighboring carbon fibers needs to be considered for clarifying the failure process of transverse cracking.

## Conclusion

In this study, a single carbon fiber transversely embedded in a dumbbell-shaped epoxy samples was observed using SR X-ray CT to detect the initiation and propagation of interfacial debonding. The summary of our findings is as follows.

- In-situ observation using SR X-ray CT was realized by developing a tabletop fatigue testing machine, which allows the direct application of both static and cyclic loads on the beamline.
- Nano-CT successfully detected interfacial debonding and indicated the formation of nano-voids in the epoxy matrix along the interface.
- The propagation of interfacial debonding between a carbon fiber and epoxy matrix under cyclic loadings can be successfully captured using transmission nano-imaging to prevent sample damage due to X-ray irradiation.

## Data Availability

The data sets used and/or analyzed within the current study are available from the corresponding author upon reasonable request and with the approval of the research sponsor.
